# Surface Water Intrusion, Land Use Impacts, and Bacterial Community Composition in Shallow Groundwater Wells Supplying Potable Water in Sparsely Populated Areas of a Boreal Region

**DOI:** 10.1128/Spectrum.00179-21

**Published:** 2021-11-03

**Authors:** Kevin J. Lyons, Anna-Maria Hokajärvi, Jenni Ikonen, Ari Kauppinen, Ilkka T. Miettinen, Tarja Pitkänen, Pekka M. Rossi, Katharina Kujala

**Affiliations:** a University of Oulu, Water, Energy and Environmental Engineering Research Unit, Oulu, Finland; b Finnish Institute for Health and Welfare, Expert Microbiology Unit, Kuopio, Finland; c University of Helsinki, Faculty of Veterinary Medicine, Department of Food Hygiene and Environmental Health, Helsinki, Finland; University of Michigan-Ann Arbor

**Keywords:** groundwater, bacteria, drinking water, microbial diversity, potable water, 16S rRNA, wells, rural, water supply, water quality, isotopes, groundwater bacteria

## Abstract

Rural communities often rely on groundwater for potable water supply. In this study, untreated groundwater samples from 28 shallow groundwater wells in Finland (<10 m deep and mostly supplying untreated groundwater to <200 users in rural areas) were assessed for physicochemical water quality, stable water isotopes, microbial water quality indicators, host-specific microbial source tracking (MST) markers, and bacterial community composition, activity, and diversity (using amplicon sequencing of the 16S rRNA gene and 16S rRNA). Indications of surface water intrusion were identified in five wells, and these indications were found to be negatively correlated, overall, with bacterial alpha diversity (based on amplicon sequencing of the 16S rRNA gene). High levels of turbidity, heterotrophs, and iron compromised water quality in two wells, with values up to 2.98 nephelometric turbidity units (NTU), 16,000 CFU/ml, and 2,300 μg/liter, respectively. Coliform bacteria and general fecal indicator *Bacteroidales* bacteria (GenBac3) were detected in 14 and 10 wells, respectively (albeit mostly at low levels), and correlations were identified between microbial, physicochemical, and environmental parameters, which may indicate impacts from nearby land use (e.g., agriculture, surface water, road salt used for deicing). Our results show that although water quality was generally adequate in most of the studied wells, the continued safe use of these wells should not be taken for granted.

**IMPORTANCE** Standard physicochemical water quality analyses and microbial indicator analyses leave much of the (largely uncultured) complexity of groundwater microbial communities unexplored. This study combined these standard methods with additional analyses of stable water isotopes, bacterial community data, and environmental data about the surrounding areas to investigate the associations between physicochemical and microbial properties of 28 shallow groundwater wells in Finland. We detected impaired groundwater quality in some wells, identified potential land use impacts, and revealed indications of surface water intrusion which were negatively correlated with bacterial alpha diversity. The potential influence of surface water intrusion on groundwater wells and their bacterial communities is of particular interest and warrants further investigation because surface water intrusion has previously been linked to groundwater contamination, which is the primary cause of waterborne outbreaks in the Nordic region and one of the major causes in the United States and Canada.

## INTRODUCTION

Groundwater is the world’s largest freshwater resource and is estimated to provide potable water for up to half of the global population, supplying many major cities and towns, as well as most rural areas (1–4). Shallow groundwater resources (e.g., <10 m below the land surface) are widespread globally (5) and are commonly exploited throughout the developing and developed world because they can provide reliable supplies of water in a technically and economically feasible manner (2, 6–8). However, shallow groundwater resources are particularly vulnerable to contamination, not only because of their proximity to the ground surface but also because of shortcomings in the management and maintenance of groundwater wells (9–13). Groundwater contamination is widely recognized as an important public health issue (7), but more work is needed to understand and mitigate the threats to groundwater systems in rural areas. These smaller systems often suffer from a lack of attention and resources and generally have more problems than larger systems (14–20).

Threats to the safe use of shallow groundwater wells for potable water supply can arise through contamination events that often correlate with changes in the physicochemical and/or microbial parameters of the water. Physicochemical changes may be caused by, for example, the influence of nearby ditches, sand or gravel pits, or salted roads in wintertime (21–24), whereas microbial contamination is typically caused by the introduction of pathogenic microbes from animal waste and/or human sewage into the water supply from nearby agricultural activities, livestock, wild animals, septic tanks, sewage systems, or surface water sources (13, 25–35). Microbes known to be associated with groundwater contamination include (i) fecal indicator bacteria, such as Escherichia coli, intestinal enterococci, *Clostridium*, and *Bacteroides*, (ii) pathogenic bacteria, such as pathogenic strains of E. coli and some species of *Salmonella*, *Shigella*, and *Campylobacter*, (iii) pathogenic viruses, such as enterovirus, norovirus, rotavirus, hepatovirus A, and adenovirus, and (iv) protozoa, such as *Cryptosporidium* and *Giardia* (7, 36, 37).

The microbial contamination of groundwater is a widespread occurrence globally and continues to cause outbreaks of gastrointestinal illness in both developing and developed countries (11, 37, 38). It is currently the primary cause of waterborne outbreaks in the Nordic region (36, 39), as well as one of the major causes in the United States and Canada (7, 40–42). Many of these outbreaks have been associated with private or community groundwater wells in rural areas (7, 40–42). Such wells are often operated by untrained personnel (43), and in many cases the water is pumped to users without treatment, which means that good groundwater quality and an intact well structure are essential to enable safe water use (13, 30, 35, 44–46). Unfortunately, these conditions are not always guaranteed, and outbreaks can arise due to poor well construction, insufficient depth of protective layer above the water table, floods and surface runoffs, fissures in bedrock, or the leakage and blockage of nearby wastewater pipes (13, 33, 34, 44–48).

Many studies of groundwater microbiology focus largely on the detection of indicator microbes such as E. coli and coliform bacteria and how these might indicate potential risks to human health (13, 31, 49–51). Recently, however, the composition, activity, and diversity of microbial communities in groundwater are being more thoroughly investigated and understood (52–66). As a result, it is becoming clear that groundwater and groundwater wells should not be treated as inert systems but rather as complex ecosystems containing a wide variety of (often uncultured) microbes that interact with each other and their environment in ways that are not yet fully known. These interactions may have implications for the management of these wells and for ensuring good drinking water quality for the people who rely on them.

The aim of our study was to examine the physicochemical, microbial, and environmental differences between shallow groundwater wells in rural areas, with the goal of discovering associations which may have implications for detecting and mitigating contamination. Our principal hypothesis was that nearby land use and/or nearby hydrology and hydrogeology (e.g., streams, lakes, bogs, and fens) would be the major factors influencing the water quality and diversity of bacterial communities in the groundwater wells. We explored this hypothesis by analyzing physicochemical and microbial data from untreated groundwater samples, stable water isotope data from nearby surface water sources, and site-specific environmental data gathered via maps and on-site evaluations.

## RESULTS AND DISCUSSION

### Variation in physicochemical parameters between wells indicates potential links with environmental data.

**FIG 1:**
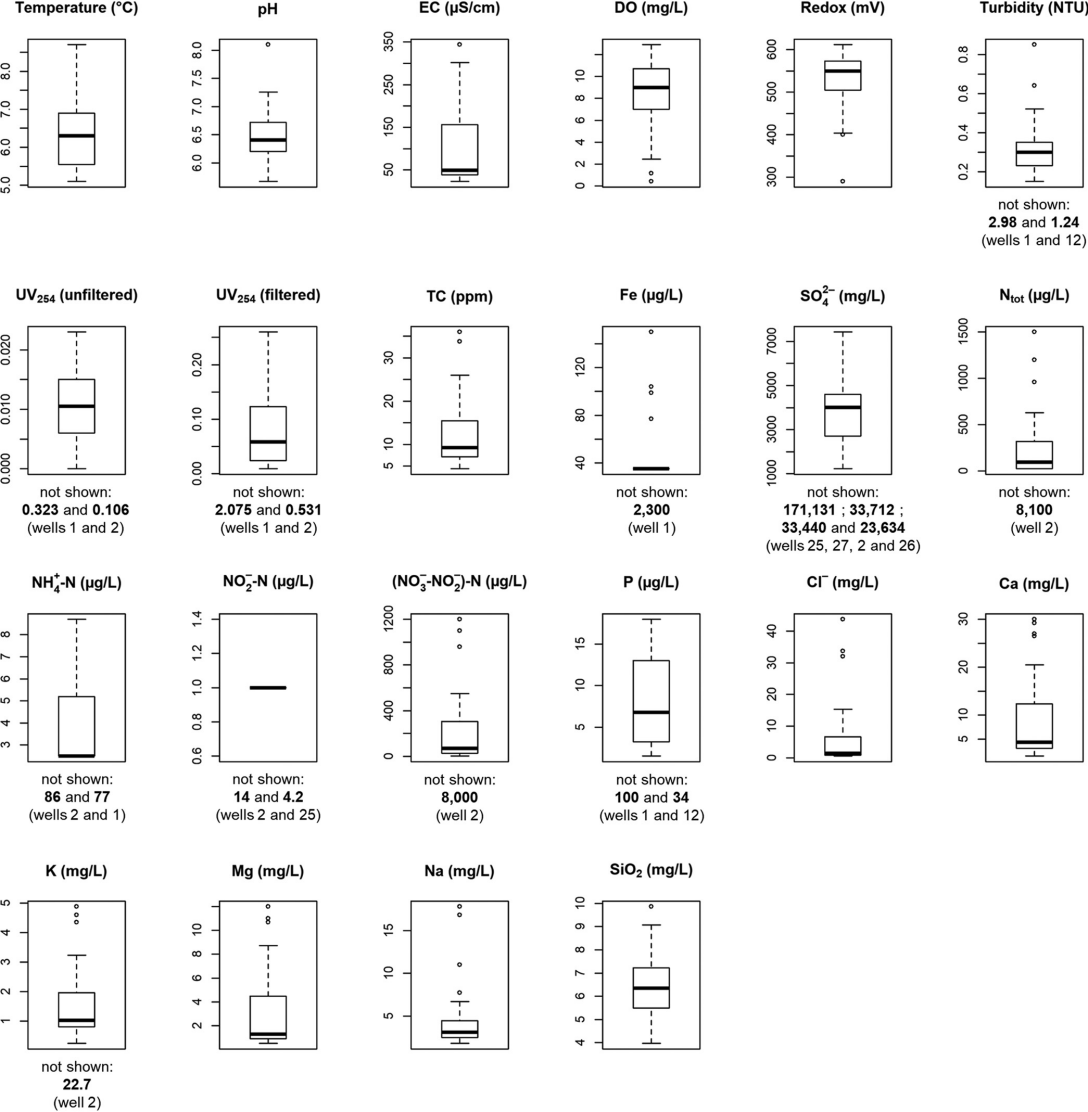
Boxplots showing physicochemical data for the 28 shallow groundwater wells. Some relatively extreme values were removed to improve plot readability. These values are indicated below the plots from which they were removed, with the corresponding well numbers given in respective order. Medians were not significantly affected by removal of these values. Boxplots were generated in R.

The groundwater from most wells was oligotrophic (low in nutrients such as N and P), with high levels of dissolved oxygen (DO; median: 9 mg/liter) and slightly acidic pH (median: 6.4) (Fig. 1). Some wells had high pH (up to 8.1) because of alkalinization material in the wells (e.g., well 21) or because of naturally high levels of Ca and Mg in the water (e.g., well 23). Well 1 stood out as having the highest overall temperature (8.7°C) and turbidity (2.98 nephelometric turbidity units [NTU]), the highest concentrations of Fe (2,300 μg/liter) and P (100 μg/liter), and the lowest redox potential (71.5 mV) and DO (0.42 mg/liter) levels. Reddish-brown staining and slime were observed on pipes at this site, suggesting that the high Fe levels were causing high water turbidity and growth of iron-oxidizing bacteria such as *Gallionella* (29.9% of all reads in the cDNA-derived 16S amplicons from this site were attributed to this genus). Also, this well was located in a clay-rich coastal area of a kind that, in Finland, is often associated with acid sulfate soils that can leach metals like Fe (67). High P levels in this well might be explained by the fact that low DO levels can cause Fe oxides in soils and aquifers to dissolve and release adsorbed P into the water (68). Well 2 had the highest overall concentrations of total nitrogen (N_tot_; 8,100 μg/liter), ammonium nitrogen (NH_4_^+^-N; 86 μg/liter), nitrite nitrogen (NO_2_^−^-N; 14 μg/liter), combined nitrate and nitrite nitrogen [(NO_3_^−^+NO_2_^−^)-N; 8,000 μg/liter], and K (22.7 mg/liter), as well as the largest nearby field area (72 ha within 1 km^2^ of the well), suggesting that high input of these nutrients may be coming from nearby agriculture (69). Well 25 had the highest concentrations of both sulfate (SO_4_^2−^; 171 mg/liter) and silica (SiO_2_) (9.86 mg/liter). The SO_4_^2−^ may have come from sulfur-containing fertilizers used in nearby agriculture (the well had the fourth largest nearby field area) or from the weathering of rocks and minerals, which are also potential sources of SiO_2_ (70, 71). Several wells also showed comparatively high concentrations of Na and chloride (Cl^−^), potentially indicating the infiltration of surface waters carrying road salt components into wells near major roads (23), as sodium chloride (NaCl) is the main road salt used in Finland. The strongest example of this was well 26, which had the highest concentrations of both Na (17.8 mg/liter) and Cl^−^ (43.7 mg/liter), as well as the largest total nearby road length (8,074 m within 1 km^2^ of the well).

### Indications of surface water intrusion identified in five wells.

**FIG 2:**
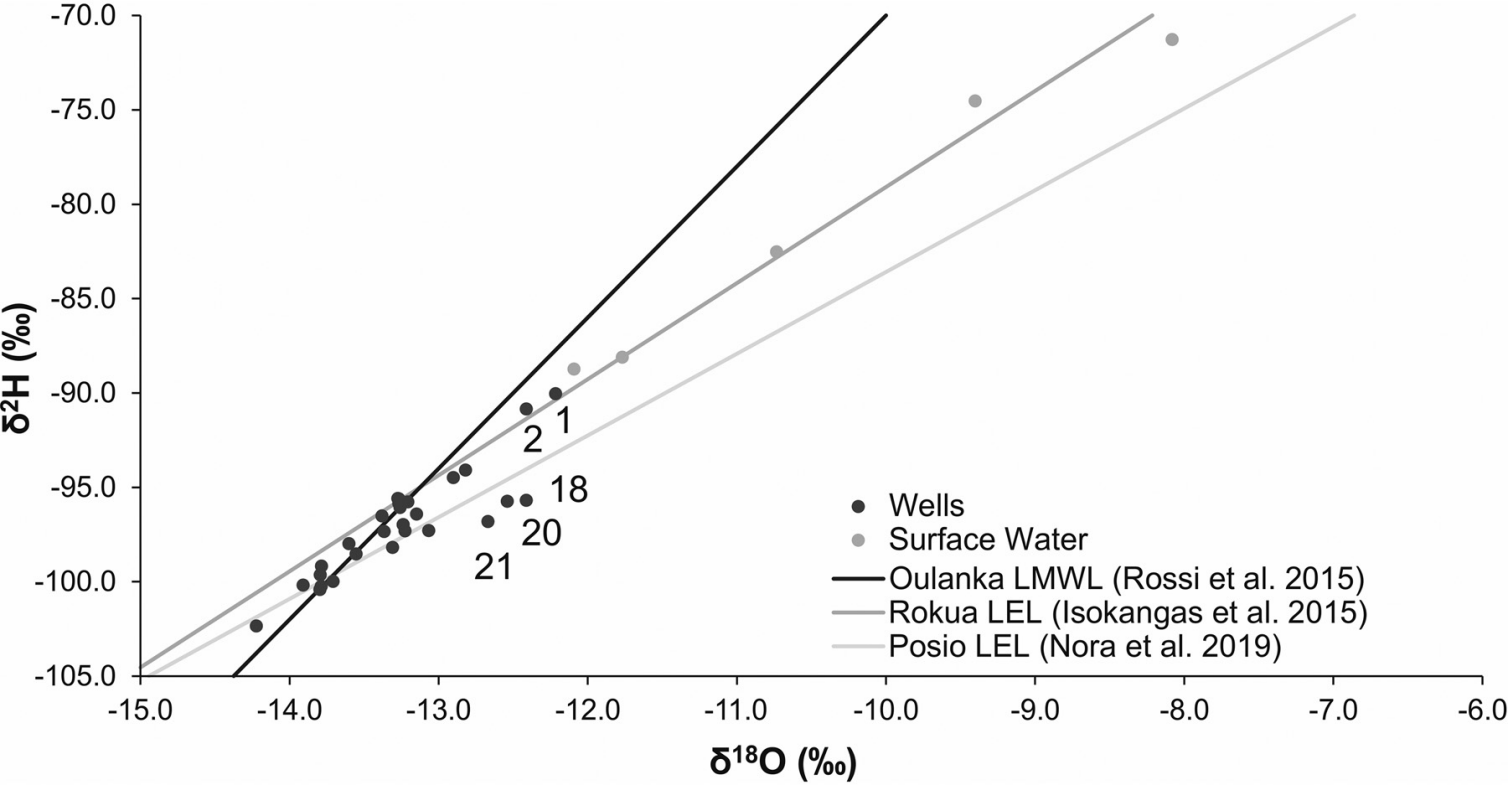
Stable water isotope results from the wells compared to surface water samples and to rainfall. Data for Oulanka local meteoric water line (LMWL) and Rokua and Posio local evaporation lines (LEL) were taken from previous studies (72, 73, 99). Wells with various d-excess values and/or alignment with LEL lines are marked with well numbers.

Most groundwater samples taken from the wells had stable water isotopes in the vicinity of the local rainfall line (i.e., the Oulanka local meteoric water line [LMWL]) (72), with δ^18^O values varying between −14.2 and −12.8, δ^2^H values varying between −102.3 and −94.1, and d-excess values varying between 7.3 and 11.4 (Fig. 2). This indicated that the source of the water in these wells was local precipitation and especially snowmelt. These are the main expected sources of groundwater recharge, and therefore the isotope signals of most wells were typical of groundwater. Contrastingly, the collected surface water samples mainly followed the Rokua local evaporation line (LEL) (73). Wells 1 and 2, which were previously identified as exceptional based on their physicochemical data, were exceptional here too. Groundwater samples taken from these wells deviated from the bulk of the samples by having stable water isotopes that followed the Rokua LEL rather than the Oulanka LMWL, with δ^2^H values of −90 and −90.8 and d-excess values of 7.7 and 8.5, respectively. Groundwater samples from wells 18, 20, and 21 were also exceptional, following the Posio LEL rather than the Oulanka LMWL, with d-excess values varying between 3.6 and 4.6 (a range different from that of the other wells). The surface water evaporation signal, the LEL, can vary regionally, depending on local conditions. For this reason, wells 18, 20, and 21 were closer to Posio LEL than to Rokua LEL. Wells 18 and 20 were relatively unremarkable based on physicochemical data, but well 21 had above average pH, Ca, electrical conductivity (EC), and total carbon (TC) levels. Overall, wells 1, 2, 18, 20, and 21 appear to have indications of surface water intrusion based on the evaporation signal in stable water isotopes. Although well 18 had the largest nearby surface water area of all wells (29 ha within 1 km^2^ of the well), “total nearby surface water area” (median: 3.54 ha within 1 km^2^ of the well) alone did not seem to be an important predictor of surface water indication in stable water isotopes. Neither did “distance to nearest surface water” (median: 80 m), with only one of the five identified wells (well 18) having a below-median value (55 m). Risk for surface water intrusion into these wells may therefore depend more on the surrounding hydrogeological conditions and precipitation patterns and/or factors relating to well construction and maintenance. Stable water isotopes have previously been used to identify surface water intrusion into wells (74), but given that the groundwater samples analyzed here represent only a single time point, repeated or continuous sampling campaigns would be needed to better understand the dynamics of the surface and well waters at these sites.

### Microbiological water quality was impaired in some wells.

Low microbial loads were detected in most wells (Table 1). The median total heterotrophic plate count was 125 CFU/ml, although some wells, such as well 2 and well 21, had comparatively high counts (1,200 CFU/ml and 16,000 CFU/ml, respectively), possibly related to surface water indications observed in stable water isotopes. E. coli was not detected in any of the wells, and virtually no coliphages or spores of sulfite-reducing clostridia were detected either, except for a very low level of F-specific coliphages (0.04 PFU/liter) in well 2 and an observation of clostridia (1 CFU/100 ml) in well 1. Coliform bacteria were detected in half of the wells, but mostly at low levels (<20 CFU/liter), with the highest levels being in well 17 (260 CFU/liter) and well 2 (80 CFU/liter). General fecal indicator *Bacteroidales* bacteria (GenBac3) were detected in DNA extracts from 3 wells (wells 1, 2, and 15) and in cDNA extracts from 10 wells (wells 1, 2, 7, 8, 15, 16, 20, 21, 24, and 26), but no human-specific fecal indicator *Bacteroides* bacteria (HF183) were detected. Wells 1, 2, and 15 also had above-median levels of NH_4_^+^-N, P, coliform bacteria, and buildings within 200 m distance from the well. Gene copies of Gram-negative bacteria were prevalent in all wells, although the RNA copy numbers of Gram-negative bacteria remained below the relatively high limit of detection in eight wells. Well 2, which exhibited relatively high levels of nutrients in the physicochemical analyses and a surface water signal in stable water isotopes, was also the most exceptional well here in terms of microbial findings, exhibiting the highest levels of 16S rRNA gene copies (0.08 genome copies [GC]/ml) and rRNA copies (2.6 GC/ml) of *Bacteroidales*, the highest levels of 16S rRNA gene copies (2,500 GC/ml) and rRNA copies (370,000 GC/ml) of Gram-negative bacteria, and the second-highest level of heterotrophic plate counts (1,200 CFU/ml).

**TABLE 1:** Summary of microbial indicators in the 28 studied groundwater wells^*a*^

Microbial indicator	Min	Median	Max	Well no. (relatively extreme values)^*b*^
E. coli (CFU/100 ml)	0	0	0	Indicator not detected
Coliform bacteria^*c*^ (CFU/liter)	0	0	260	Well 17 (260), well 2 (80)
Coliform bacteria^*d*^ (CFU/liter)	0	0	210	Well 17 (210), well 2 (80)
SSRC (CFU/100 ml)	0	0	1	Well 1 (1)
Heterotrophic bacteria (CFU/ml)	5	125	16,000	Well 21 (16,000), well 2 (1,200)
Somatic coliphages (PFU/liter)	0	0	0	Indicator not detected
F-specific coliphages (PFU/liter)	0	0	0.04	Well 2 (0.04)
*Bacteroidales* rRNA gene (GenBac3) (GC/100 ml)	0	0	8	Well 2 (8), well 1 (7), well 15 (4)
*Bacteroidales* rRNA (GenBac3) (GC/100 ml)	0	0	260	Well 2 (260), well 15 (183), well 14 (81), well 8 (67)
*Bacteroides* rRNA gene (HF183) (GC/100 ml)	0	0	0	Indicator not detected
*Bacteroides* rRNA (HF183) (GC/100 ml)	0	0	0	Indicator not detected
Gram-negative bacteria (rRNA gene) (GC/100 ml)	1,400	13,000	250,000	Well 2 (250,000), well 14 (100,000), well 1 (96,000)
Gram-negative bacteria (rRNA) (GC/100 ml)	0	450,000	37,000,000	Well 2 (37,000,000), well 21 (9,700,000)

a SSRC, spores of sulphite-reducing Clostridia; GC, genome copies.

b Sites with values greater than one standard deviation above the median.

c SFS 3016 method.

d ISO 9308-1 method.

### Differences observed in alpha diversity metrics of bacterial DNA- and cDNA-derived 16S amplicons.

**FIG 3:**
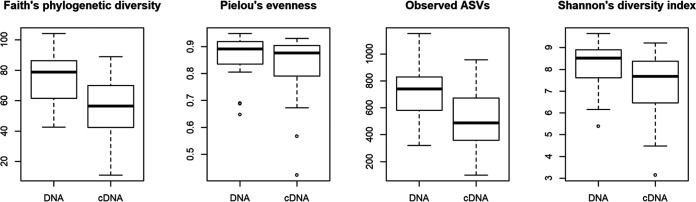
Boxplots showing differences between alpha diversity metrics in DNA- and cDNA-derived 16S amplicons. Boxplots were generated in R.

Bacterial 16S rRNA amplicons were sequenced from DNA and cDNA. On average, approximately 35,400 quality-filtered sequences were obtained per library. The median number of observed amplicon sequence variants (ASVs) and median values for Faith’s phylogenetic diversity and Shannon diversity were lower for the cDNA libraries than for the DNA libraries (Fig. 3 and Table 2). The lower diversity in cDNA libraries may indicate a selective activation of bacterial taxa (i.e., not all present taxa are equally active and a small number of active taxa dominate), or it may simply be a reflection of the fact that DNA persists longer than RNA in natural waters, such that DNA-based diversity is better preserved.

**TABLE 2:** Summary of alpha diversity metrics

Alpha diversity metric	Min	Median	Max	Well no. (relatively extreme values)^*a*^	
				High	Low
Faith’s PD (DNA)	42.6	78.81	104.2	Well 9 (104.24), well 23 (94.56)	Well 2 (42.63), well 21 (48.23), well 27 (55.03), well 16 (57.22), well 18 (59.87), well 7 (60.23), well 15 (60.39), well 22 (62.57)
Faith’s PD (cDNA)	11.1	56.54	89.04	Well 23 (89.04), well 26 (88.00), well 5 (79.86), well 20 (79.29), well 10 (77.86)	Well 21 (11.05), well 12 (21.11), well 2 (22.18), well 14 (23.35), well 1 (24.64), well 15 (33.27)
Pielou’s evenness (DNA)	0.65	0.89	0.95		Well 2 (0.65), well 22 (0.69), well 15 (0.69), well 21 (0.81)
Pielou’s evenness (cDNA)	0.42	0.88	0.93		Well 12 (0.42), well 15 (0.57), well 2 (0.67), well 21 (0.67), well 28 (0.71)
Observed ASVs (DNA)	321	742	1153	Well 9 (1153), well 8 (971), well 17 (942)	Well 2 (321), well 21 (404), well 27 (451), well 22 (492), well 16 (500), well 18 (538)
Observed ASVs (cDNA)	100	489	957	Well 26 (957), well 20 (831), well 23 (819), well 10 (809), well 5 (807), well 3 (718)	Well 21 (100), well 12 (174), well 14 (174), well 2 (184), well 1 (227)
Shannon’s diversity (DNA)	5.39	8.52	9.65	Well 9 (9.65)	Well 2 (5.39), well 22 (6.15), well 15 (6.33), well 21 (6.98), well 27 (7.40), well 18 (7.41), well 16 (7.44)
Shannon’s diversity (cDNA)	3.15	1.53	9.21	Well 26 (9.21)	Well 12 (3.15), well 21 (4.48), well 15 (4.63), well 2 (5.06), well 14 (5.92), well 28 (5.98), well 1 (6.1)

a Sites with values greater than one standard deviation above or below the median.

Wells 2 and 21 were exceptional here again, being characterized by relatively low values of all four alpha diversity metrics in both DNA and cDNA libraries. This is possibly related to surface water influence noted by the signal in stable water isotopes. The relatively high levels of heterotrophic bacteria and gene copy numbers of Gram-negative bacteria in wells 2 and 21, together with low alpha diversity values, suggest that, at the time of sampling, the bacterial communities in these wells were dominated by a limited group of active bacteria. Wells 9, 23, and 26 had relatively high levels of at least three alpha diversity metrics.

### Different dominant taxa in DNA and cDNA libraries and some exceptional wells.

**FIG 4:**
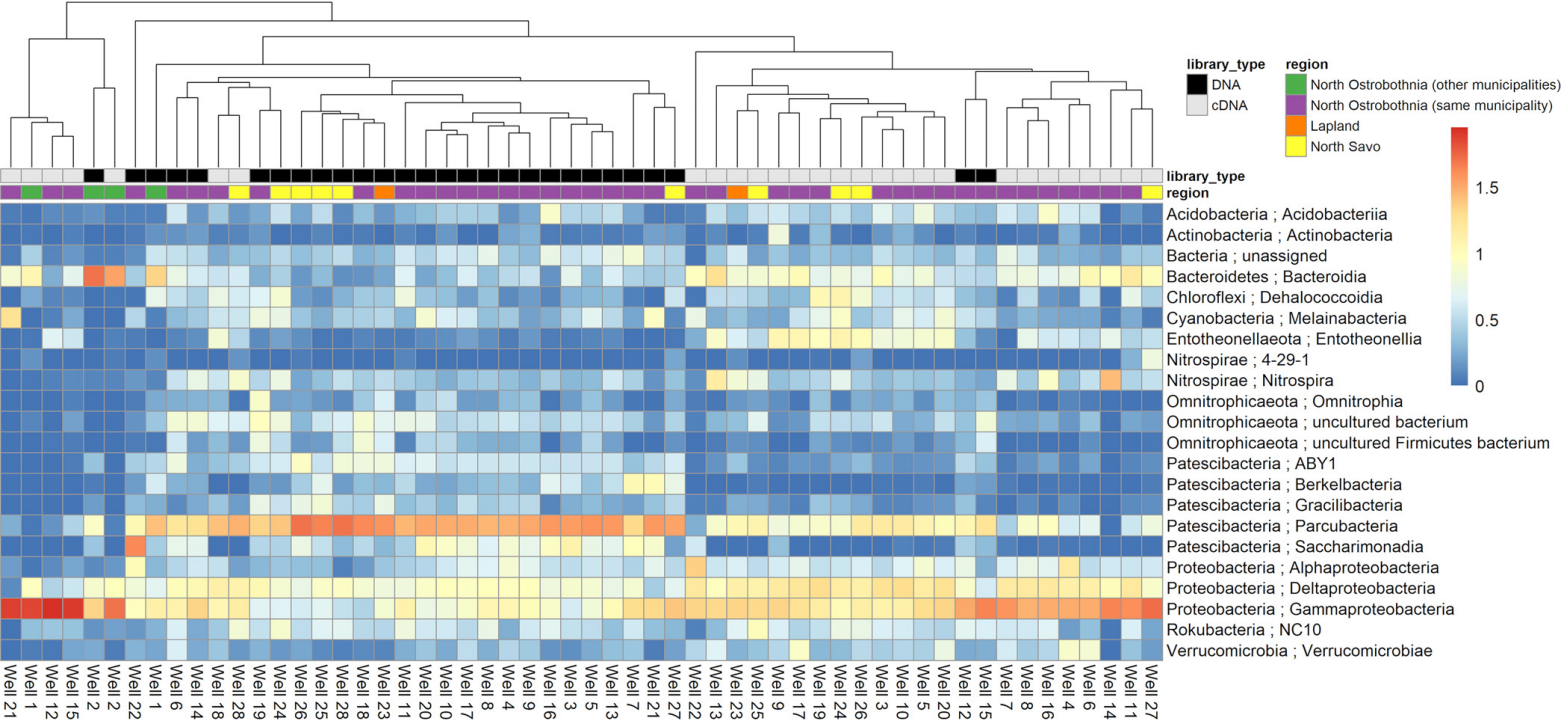
Heatmap showing differences in bacterial communities based on taxonomic classifications of DNA- and cDNA-derived 16S amplicons generated in QIIME 2 using the SSU SILVA 132 majority taxonomy. The heatmap was generated in R (using the pheatmap package) (113) from the log-transformed relative abundance values of bacterial classes which had a relative abundance of 5% or more in at least one library. Columns were clustered using average linkage hierarchical clustering based on the Bray–Curtis dissimilarity matrix of the data set (using the vegan package) (116).

Taxonomic classification of 16S sequences revealed differences between DNA and cDNA libraries (Fig. 4). The most commonly identified bacterial taxa in the DNA-derived 16S amplicons—based on mean relative abundance values of phyla, with *Proteobacteria* split to the class level—were *Patescibacteria* (43.5%), *Gammaproteobacteria* (11.5%), *Omnitrophicaeota* (7.5%), Deltaproteobacteria (6.6%), and *Bacteroidetes* (4.5%). Members of the *Patescibacteria* superphylum have previously been shown to dominate DNA-derived 16S rRNA gene amplicons in groundwater environments (58, 59, 61, 75). These bacteria have particularly small cell sizes and are not easily cultivated (56). The most commonly identified taxa in the cDNA libraries were *Gammaproteobacteria* (31.9%), *Deltaproteobacteria* (11.8%), *Patescibacteria* (8.4%), *Bacteroidetes* (8.0%), and *Entotheonellaeota* (4.4%). The most commonly identified taxa in the control libraries were *Firmicutes* (32.1%), *Gammaproteobacteria* (28.8%), *Actinobacteria* (15.8%), *Bacteroidetes* (11.8%), and *Cyanobacteria* (2.3%). In 20 of the wells, *Patescibacteria* were dominant in DNA libraries and *Gammaproteobacteria* were dominant in cDNA libraries. However, three wells (wells 2, 12, and 15) had DNA libraries with >10% higher relative abundances of *Gammaproteobacteria* than of *Patescibacteria*, combined with above-median numbers of coliform bacteria, heterotrophic plate counts, and P concentrations. Wells 2 and 15 also had above-median concentrations of NH_4_^+^-N and both DNA-derived and RNA-derived copy numbers of a general fecal *Bacteroidales* marker (GenBac3). Well 2 also had an indication of surface water intrusion in stable water isotopes. Similarly, two wells (wells 18 and 28) had cDNA libraries with >15% higher relative abundances of *Patescibacteria* than of *Gammaproteobacteria*. Both of these wells had above-median values for EC, N_tot,_ (NO_3_-+NO_2_^−^)-N, SO_4_^2−^, Cl-, Ca, K, Mg, Na, “nearby field area,” and “number of nearby buildings” and below-median values for “distance to surface water.” Well 18 also had an indication of surface water intrusion in stable water isotopes. Based on these observations, it seems as though a *Gammaproteobacteria*-*Patescibacteria* ratio of >1 in DNA libraries or <1 in cDNA libraries could perhaps serve as some kind of water quality indicator in these wells, although further work would be needed to verify this. Escherichia coli was not detected in any of the DNA or cDNA libraries, nor were any of the other bacterial pathogens which were screened in this study.

There was also some evidence that bacterial taxa with certain metabolic lifestyles are found at higher relative abundance in wells with suitable physicochemical properties. For example, well 1, which had the highest levels of Fe, had one of the highest relative abundances of the *Gallionella* genus of iron-oxidizing bacteria in DNA (29.9%) and cDNA (6%) libraries. Well 25, which had the highest levels of SO_4_^2−^, had the highest relative abundances of *Beggiatoaceae* DNA (1.14%) and cDNA (11.14%), a family of sulfur-oxidizing bacteria. In addition, some wells with high levels of *Bacteroidales* in quantitative PCR (qPCR) and reverse transcriptase quantitative PCR (RT-qPCR) also had high relative abundances of *Bacteroidetes* in 16S rRNA gene and rRNA libraries. For example, well 2 had the highest levels of *Bacteroidales* DNA (80 GC/liter) and cDNA (2,600 GC/liter) in (RT)-qPCR and the highest relative abundance of *Bacteroidetes*, the phylum containing the order *Bacteroidales*, in DNA (51.2%) and cDNA (35.3%) libraries. It is worth noting, however, that due to the compositional nature of 16S rRNA amplicon sequencing data, the absolute abundances of the bacteria identified by 16S rRNA amplicon sequencing remain unknown (76).

DNA- and cDNA-derived 16S amplicons largely clustered apart, indicating general differences between “present” (DNA) and metabolically “active” (cDNA) bacterial communities. Differences between present and active microbial communities are often observed in other studies (61, 64, 77). However, use of amplicon sequencing of the 16S rRNA to approximate the active fraction of a bacterial community has its limitations (78).

Some of the cDNA libraries formed a small outlier group during clustering (far left of Fig. 4), with noticeably higher relative abundances of *Gammaproteobacteria* and lower relative abundances of *Parcubacteria* than those of the other cDNA libraries. Some of these exceptional libraries were from wells previously identified as exceptional based on physicochemical data, microbiological analysis results, stable water isotopes, or *Gammaproteobacteria*-*Patescibacteria* ratio: wells 1, 2, 12, 15, and 21, for example. Such abnormalities may warrant further investigation of potential risks to the continued use of these wells for potable water supply.

### Physicochemical and microbial correlations and potential land use impacts.

**FIG 5:**
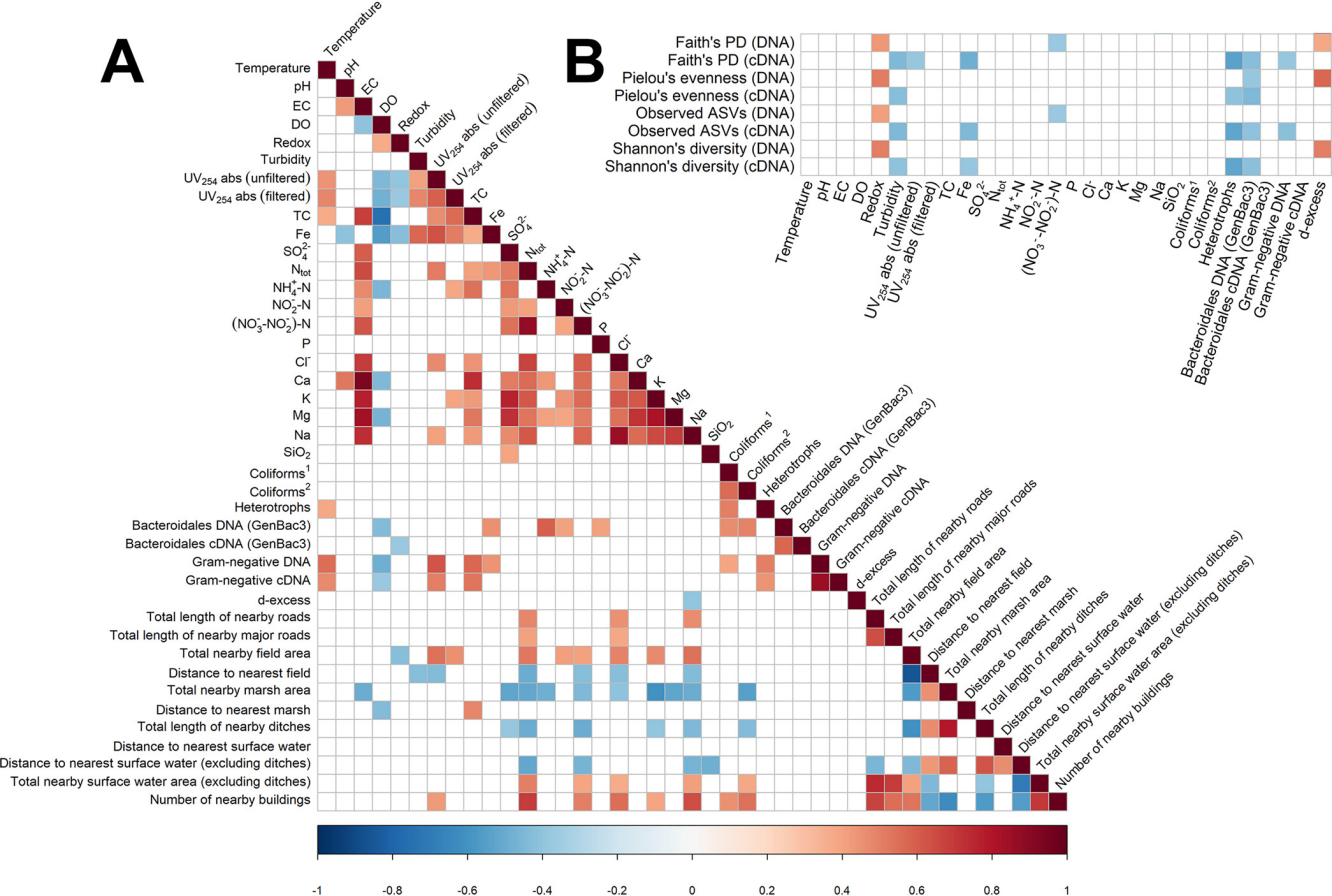
Correlograms showing (A) correlations between physicochemical data, microbiological data, and environmental data and (B) correlations between physicochemical data and alpha diversity metrics. Both correlograms were constructed using a Spearman rank-based correlation coefficient matrix and associated *P* values. Only the statistically significant correlations (*P* < 0.05) are shown. Red colors are positive correlations. Blue colors are negative correlations. In each case, the intensity of the color indicates the strength of the correlation. Spearman rank-based correlation coefficients were calculated using the rcorr function from the Hmisc R package (114), and correlograms were produced using the corrplot function from the corrplot R package (115). ^1^SFS 3016 method, ^2^ISO 9308-1 method.

Many statistically significant correlations (*P* < 0.05) were identified among and between physicochemical and microbial parameters (Fig. 5A). Fe had a strong positive correlation with UV_254_ absorbance of unfiltered water (0.64) and a moderate positive correlation with turbidity (0.58). Turbidity can be caused by clay, silt, nonliving organic particulates, plankton, microbes, or suspended organic or inorganic matter. Turbidity caused by suspended inorganic matter is particularly common in groundwater, and precipitated iron oxides/hydroxides are one source (as was visibly observed at well 1) (43, 79, 80). Turbidity is known to influence absorbance throughout the UV spectrum (81, 82), and high positive correlation coefficients have previously been reported between turbidity and UV_254_ absorbance (83).

There was a strong negative correlation between DO and TC (−0.74), which may be related to the fact that heterotrophic microbes typically consume organic carbon most efficiently via aerobic respiration. There were positive correlations between TC and Gram-negative DNA (0.57) and cDNA (0.54) and between UV_254_ absorbance of unfiltered water and Gram-negative DNA (0.63) and cDNA (0.51). TC and UV_254_ absorbance of unfiltered water were also positively correlated with each other (0.57). This is unsurprising, perhaps, as UV_254_ absorbance is an indicator for total organic carbon (TOC) and dissolved organic carbon (DOC), higher levels of which enable better growth of bacteria in water (84–86). Positive correlation coefficients have been previously reported in groundwater for bacterial colony counts and UV_254_ absorbance (87) and for DOC and Gram-negative bacteria such as coliforms (88).

“Total length of nearby roads” was positively correlated with N_tot_, Cl^−^, and Na, the first of which may originate from vehicle emissions and the latter two from road salt (23, 89). “Total nearby field area” was positively correlated with Na (0.54), UV_254_ absorbance of unfiltered water (0.54), N_tot_ (0.52), Cl^−^ (0.50), K (0.48), NO_2_^−^-N (0.42), and (NO_3_^−^+NO_2_^−^)-N (0.40), many of which may be linked to fertilizer use (90–92). Roads and fields were not correlated with microbial data.

“Distance to nearest surface water (excluding ditches)” was negatively correlated with N_tot_ (−0.52), (NO_3_^−^+NO_2_^−^)-N (−0.47), and Na (−0.47), and “total nearby surface water area” was positively correlated with the same chemicals (0.50, 0.42, 0.40), suggesting that surface waters may be a potential source of these chemicals in the groundwater wells. “Number of nearby buildings” was positively correlated with N_tot_ (0.69), Na (0.65), Cl^−^ (0.55), coliforms (0.55 and 0.46), (NO_3_^−^+NO_2_^−^)-N (0.50), and UV_254_ absorbance of unfiltered water (0.43). N_tot_, Na, and Cl^−^ might appear here, partly because buildings and roads are positively correlated (0.67 and 0.55). The limitations of correlation analysis are quite visible here, as roads, surface water area, buildings, and fields are mostly all positively correlated with one another, making it difficult to determine the exact sources of various chemical and microbial parameters. No statistically significant correlations were identified between environmental data and alpha diversity metrics, suggesting that the environmental data collected in this study were not sufficient to explain differences in bacterial alpha diversity between wells (Table S1).

### Bacterial alpha diversity correlated negatively with surface water intrusion and positively with redox potential.

Several statistically significant correlations (*P* < 0.05) were identified between the physicochemical data and alpha diversity metrics (Fig. 5B). For the DNA-derived 16S rRNA gene amplicons, redox potential had positive correlations with all four alpha diversity metrics (range: 0.42 to 0.52), and d-excess had positive correlations with Pielou’s evenness (0.57), Shannon’s diversity (0.51), and Faith’s phylogenetic diversity (PD; 0.38). Low d-excess values are indicative of surface water intrusion, so intrusion appears to be associated with relatively lower bacterial diversity in the groundwater wells. Further work would be needed to test causation, but perhaps intrusion can cause reduced diversity by introducing generalist bacteria or fresh surface materials from surface water and soil into the naturally occurring, largely oligotrophic groundwater community (93). Microbial communities in groundwater have been shown to react sensitively to surface water intrusion in the context of riverbank filtration, with losses of resident taxa indicated by declining alpha diversity (66), similar to the low diversity found here. However, by contrast, a recent study of a fractured bedrock aquifer found that proximity to the recharge area gave prominence to high bacterial diversity, with the authors proposing that this high diversity was largely due to episodic input of surface soil-derived bacteria (65). Thus, the influence of surface water intrusion on bacterial diversity may be site dependent, or otherwise variable, and warrants further investigation. Yan et al. studied a series of wells at different points on a single hillslope above a fractured bedrock aquifer in the temperate broadleaf biome (65), whereas the wells studied here are largely from sand and gravel aquifers at different locations within the boreal biome, mostly with relatively flat surrounding topographies. These and other differences (e.g., precipitation rates, exact aquifer type and structure, recharge rates, well structure and maintenance) may give rise to different associations between surface water intrusion and bacterial diversity. Microbial communities catalyze important biogeochemical processes in groundwater, such as the turnover of carbon and other nutrients, as well as pollutant attenuation (57, 94), so disturbances and changes to their community composition via surface water intrusion may have important implications for drinking water quality and safety, and thereby for the proper management of groundwater wells, given that surface water intrusion has previously been identified as a direct risk factor for waterborne outbreaks (13, 39, 47).

For the cDNA libraries, turbidity, heterotrophs, and *Bacteroidales* DNA (GenBac3) had moderate negative correlations with all four alpha diversity metrics (ranges: −0.44 to −0.40, −0.40 to −0.54, and −0.45 to −0.41), Fe had weak to moderate negative correlations with Faith’s phylogenetic diversity, observed ASVs, and Shannon’s diversity (range: −0.48 to −0.38), Gram-negative DNA had weak to moderate negative correlations with Faith’s phylogenetic diversity and observed ASVs, and UV_254_ absorbance of unfiltered water had a moderate negative correlation with Faith’s phylogenetic diversity (−0.39).

### Nonmetric multidimensional scaling.

**FIG 6:**
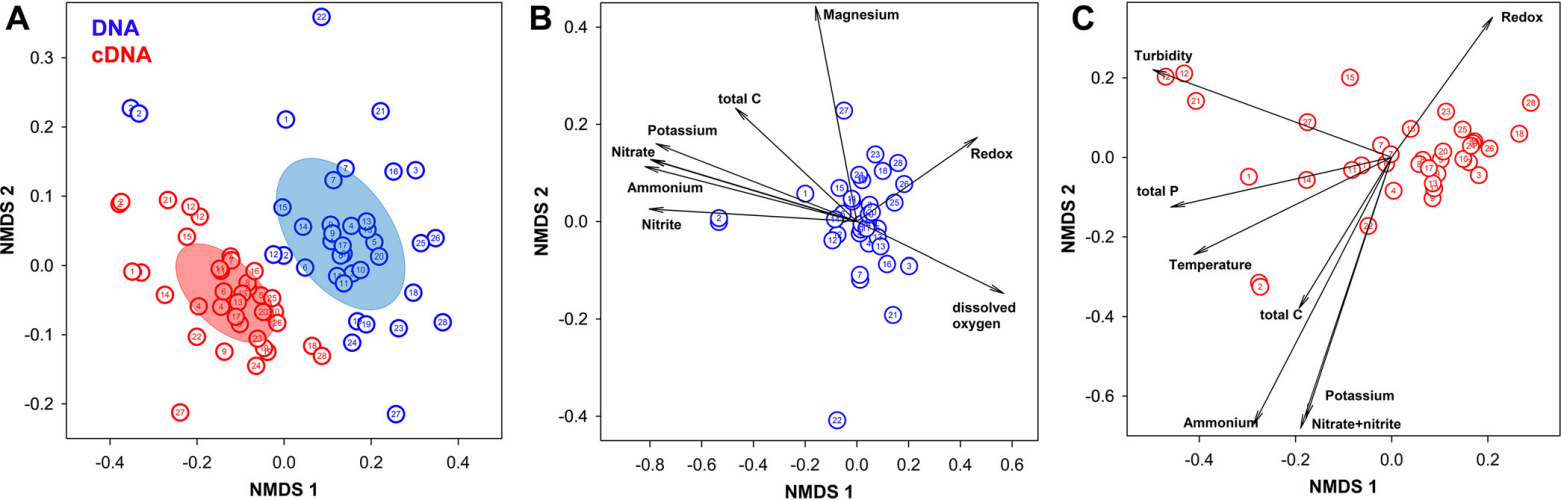
Nonmetric multidimensional scaling (NMDS) based on sequence analysis of bacterial 16S rRNA on the DNA (blue) and cDNA (red) levels. Comparison of bacterial communities on the DNA and cDNA levels (A) and effect of environmental parameters on bacterial community composition on the DNA (B) and cDNA (C) levels. In panel A, dispersion ellipses indicate centroids of microbial communities on the DNA and cDNA levels. In panels B and C, selected environmental parameters (*P* ≤ 0.05) fitted to the ordinations are indicated by arrows. Well numbers are indicated inside the data points.

Nonmetric multidimensional scaling (NMDS) based on sequence analysis of bacterial 16S rRNA on the DNA and cDNA level revealed overall differences between DNA- and cDNA-based bacterial communities (Fig. 6A). In addition, fitting of environmental parameters to the NMDS plots for DNA- and cDNA-based bacterial communities revealed that several environmental parameters correlated significantly with bacterial communities (Fig. 6B and C). The compositions of DNA- and cDNA-based communities are influenced by redox potential, total carbon, potassium, ammonium nitrogen (NH_4_^+^-N), and combined nitrate and nitrite nitrogen [(NO_3_^−^+NO_2_^−^)-N]. DNA-based communities were additionally influenced by magnesium, dissolved oxygen, and nitrite nitrogen (NO_2_^−^-N), whereas cDNA-based communities were additionally influenced by turbidity, total phosphorus, and temperature. Nutrients such as N, P, and C, and factors such as temperature, redox potential, and dissolved oxygen, are well known to influence the ability of specific bacterial taxa to survive and propagate in various ecosystems (95). As groundwater is often considered an oligotrophic (nutrient-poor) environment (93), the idea that nutrients such as N and P could influence bacterial community composition is not entirely surprising, but the possibility that nutrient inputs from agriculture or surface waters could alter bacterial community composition in these shallow groundwater wells is still worth considering in case it may have implications for maintaining a safe potable water supply.

### Revised EU Drinking Water Directive and water safety planning.

The recently revised EU Drinking Water Directive 2020/2184 promotes risk-based approaches and better transparency for drinking water consumers throughout the European Union. However, it remains to be seen how the new directive will affect the smallest water suppliers, because it does not require EU member states to carry out risk assessments on water suppliers supplying 10 to 100 m^3^ per day or serving 50 to 500 people, and water quality sampling for these supplies need be conducted only once or twice a year. Finnish national legislation requires risk assessments at even the smallest supplies, but other countries may choose to exempt these sites to reduce potential administrative burden.

Given that shallow groundwater resources are often particularly vulnerable to contamination (9–13), and that investments in interventions aimed at improving rural community water supplies are highly cost beneficial in the developed world (96), we propose that risk assessments should be more carefully considered for shallow groundwater wells, especially in cases where surface water intrusion or other risks are indicated. Such risk assessments would involve thorough sanitary surveys (water safety planning) accompanied by, if possible, detailed microbial and physicochemical investigations which include the use of novel analytical methods (e.g., analyses of stable water isotopes, biomarkers, and microbial communities). These assessments would lead to better understanding and predicting of contamination events and better aversion of potential negative health consequences through remedial actions, such as applying water treatment and decontamination and/or eliminating the contamination source(s). In the event that such detailed site-specific analyses are not possible, due to time and resource constraints, findings from this study and similar studies can serve as a starting point for interpreting potential risks to water quality in shallow groundwater wells.

### Conclusions, limitations, and outlook.

Our findings provide further evidence that groundwater wells should not be treated as inert structures but rather as complex ecosystems influenced by many factors that are not yet fully known. We pinpointed several potentially problematic wells on the basis of combined physicochemical, microbial, or environmental parameters that may be linked to various nearby water quality risk factors arising from the impacts of land use such as agriculture, roads, surface water, and other human activity. Future work will consider seasonal variation in physicochemical, microbial, and environmental parameters—something which was not assessed here—and further explore the question of surface water intrusion to better assess its risk to water quality and safety and its association with, and potential influence on, groundwater microbial communities, as well as metagenomic analysis of selected groundwater samples to investigate the functional capabilities of microorganisms in these wells.

## MATERIALS AND METHODS

### Groundwater wells and sampling methods.

**FIG 7:**
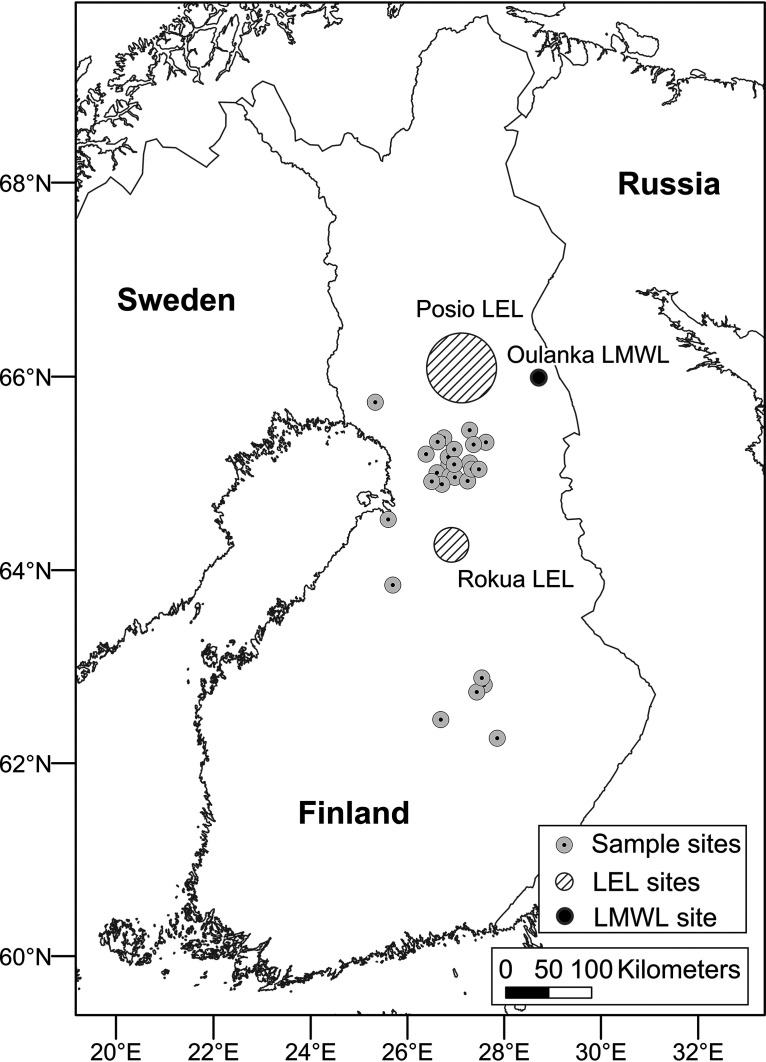
Map of the well locations and the sampling sites and regions for additional stable water isotope samples for rain (black point; Oulanka LMWL, reference 72) and surface water evaporation (lined areas; Rokua LEL, reference 73, and Posio LEL, reference 99). LMWL, local meteoric water line; LEL, local evaporation line.

Water sampling for this study was carried out during October/November 2018 at 28 shallow groundwater wells used as sources of potable water in the North Ostrobothnia, North Savo, and Lapland regions of Finland (between 66°15′ and 62°15′N, and 24°30′ and 28°30′E) (Fig. 7). The studied sites are virtually all in rural locations and have mostly flat surrounding topographies, estimated annual precipitation of 600 to 700 mm, and shallow groundwater tables (about 3 m below the land surface, on average). Twenty of the wells (wells 3 to 22) are in a single, sparsely populated municipality in North Ostrobothnia (between 65°45′ and 65°0′N, and 26°15′ and 27°45′E). Wells 1 and 2 are in other municipalities of North Ostrobothnia. Well 23 is in Lapland, and wells 24 to 28 are in North Savo. Relevant characteristics of all 28 shallow groundwater wells are shown in Table 3. Raw water samples, before any treatment processes, were collected from each well aseptically from a sampling tap into sample containers. In the case of wells 11, 13, and 21, the raw water sample was alkalized due to presence of alkalization material in the well. Prior to stable water isotope analysis, the samples were stored at 4°C. At each well, a large volume of groundwater (200 liters) was filtered by a dead-end ultrafiltration method (DEUF; ASAHI Rexeed-25A, Asahi Kasei Medical Co., Ltd., Tokyo, Japan) as described earlier by reference 77 to concentrate the otherwise highly diluted microbes for further analysis. The flow rate during DEUF sampling was adjusted to around 1 liter/18 s (3.33 liter/min), which enabled 200 liters of water to be filtered in about 1 h. Sample containers and DEUF capsules were transported in cool boxes immediately (within 24 h after sample collection) to the laboratories for physicochemical and microbiological analysis.

**TABLE 3:** Characteristics of the 28 shallow groundwater wells^*a*^

Well no.	Treatment status	Users	Water intake (m^3^/day)	Year changes to well structure last made	Well type	Well depth (m)	GW depth near well (m)	Potential nearby risk factors (within 1 km^2^)
1	UV, ALK, CH	7,000^*b*^	NA	1993	Tube	≥8	1.5	A, SW, R, RA, R, S, C
2	UV, ALK	6,400^*b*^	250	1978	Dug	6	3	A, SL
3	None	190	32.9	1984	Dug	6	2.5	M
4	None	10	1	1992	Dug	∼3	1.5	SG, M
5	ALK	40	24	1974	Dug	3	1.5	A
6	None	150	16	1986	Tube	8	4	D
7	None	<50	<5.5	NA	NA	NA	NA	M, SW, R
8	None	<100	13.7	1980s	Dug	6	2	M, A, SGP
9	None	100–200	11	1979	Dug	5	2	R, MW, SW
10	None	105	8.9	1984	Dug	5.5	2	M, D, P
11	ALK^*c*^	100	<11	1987	Dug	6	4.2	B, R, SG
12	None	∼170	12	1984	Dug	7	2	SG
13	ALK^*c*^	∼280	11	1979	Dug	5	2.5	M, P
14	None	50	8.3	1979	Dug	NA	2	M, D
15	None	150	125	1983	Dug	7	3	SW, B, SR
16	ALK	150	65	1983	Tube	11	3	WW
17	None	50–60	16.4	1984	Dug	4	1	SG, SW
18	None	155	71.2	1983	Dug	6	3.5	R, SW, SG
19	UV, ALK	4,200^*b*^	650	1961	Dug	7.5	3	SG, SA, S, R, SW
20	None	80	27.4	1984	Dug	4	∼2	M, D
21	ALK^*c*^	<50	10	1980s	Dug	3.5	1	SW, SG
22	None	28	5	1989	Tube	7	>0.5 (artesian spring)	M
23	UV, ALK	20,000^*b*^	NA	2007	Tube	7	4	M, D, SW
24	UV, ALK	1,000	170	2014	Tube	9.2	2	P, SG
25	ALK	>200	38	1990	Tube	7.5	3	A, SW, SG, SL, C
26	UV, ALK	20,000^*b*^	600–1,000	1969	Dug	9	5–10	B, R, SW, T
27	ALK	500	120	1987	Tube	NA	NA	A, SL
28	ALK	2,000	400	1988	Tube	NA	NA	R, A, SL, SG

*^a^*ALK, alkalization; UV, UV disinfection; CH, chemical purification; GW depth, groundwater depth; R, roads; SW, surface water; M, marsh; D, ditches; A, agriculture; B, buildings; P, peat production; RA, recreational area; C, cemetery; S, school; SL, slurry storage tank; SG, sand or gravel pit; MW, meltwater; WW, wastewater; SR, ski resort; SA, swimming area; T, town; NA, not applicable.

b Water served from several wells to the same network.

c Raw water samples have been alkalized.

### Physicochemical water quality analyses.

Temperature, pH, dissolved oxygen (DO), redox potential, and electrical conductivity (EC) of groundwater samples were measured on site using portable field meters, according to the manufacturer’s instructions (SenTix 940, FDO 925, SenTix ORP 900, TetraCon 325; WTW, Weilheim, Germany). Field redox potential values were converted to standard redox potential values by temperature-based adjustment. Analyses of total nitrogen (N_tot_), ammonium nitrogen (NH_4_^+^-N), nitrite nitrogen (NO_2_^−^-N), phosphorus (P), combined nitrate and nitrite nitrogen (NO_3_^−^+NO_2_^−^)-N, chloride (Cl^−^), calcium (Ca), potassium (K), magnesium (Mg), sodium (Na), and silica (SiO_2_) were performed in an accredited commercial laboratory according to international standards for chemical water quality. Iron (Fe) and sulfate (SO_4_^2−^) concentrations were determined colorimetrically via the phenanthroline method and the barium gelatin method, respectively (97, 98). Total carbon (TC) values were determined using a Sievers 900 portable TOC analyzer. Turbidity (EN 27027:1994) was determined using a Hach Ratio XR turbidity meter. UV absorbance values of unfiltered and 0.45-μm-filtered water samples were determined at 254 nm (UV_254_) using a UV-1800 spectrophotometer (Shimadzu, Japan) according to the manufacturer’s instructions.

### Stable water isotope analyses.

To identify anomalies which may indicate surface water intrusion into the groundwater wells, stable water isotope (δ^18^O, δ^2^H) analyses were conducted on untreated groundwater samples from each of the wells and on water samples taken from nearby surface water sources, such as rivers and lakes. (These well and surface water samples were collected in 15-ml high-density polyethylene tubes, which were rinsed with the sampled water before filling.) The isotope ratios ^2^H/^1^H and ^18^O/^16^O were determined using cavity ring-down spectroscopy with a Picarro L2130-i analyzer. All isotope ratios are expressed in δ notation relative to Vienna Standard Mean Ocean Water 2 (VSMOW2) with precision for δ^18^O and δ^2^H values of δ0.025‰ and δ0.1‰, respectively. The stable water isotope samples were compared to regional results for rainwater and surface water signals. A local meteoric water line (LMWL) based on data collected from the Oulanka region was used as the rainwater reference (72). For the surface water local evaporation line (LEL), the references were from the Rokua region (73) and from the Posio municipality, collected in a parallel project by the Geological Survey of Finland in 2018 (99). Surface water is prone to evaporation, which can cause deuterium isotope values to differentiate from oxygen isotope values. This deuterium excess (d-excess = δ^2^H − 8 δ^18^O) (100) was determined for the well water samples in order to study the effect of evaporative fractionation on the samples potentially resulting from surface water intrusion.

### DEUF capsule elution and coliphage analyses.

In the laboratory, DEUF capsules were eluted as described earlier (77) except that the secondary concentration of DEUF eluates of 35 to 250 ml was performed by filtration through 0.22-μm Millipore Express PLUS membrane filters (Merck KGaA, Darmstadt, Germany). Polyethylene glycol (PEG) precipitation of filtrate (200 to 500 ml) after Millipore Express PLUS membrane filtration was performed as described earlier (101), and analyses of somatic coliphages and F-specific coliphages were performed immediately from PEG precipitates using a double agar layer (DAL) procedure (USEPA Method 1601; with excess precipitate being stored at −75°C or lower). Millipore Express PLUS membranes were stored at −75°C or lower prior to nucleic acid extraction.

### Enumeration of microbial indicators.

Escherichia coli and coliform bacteria were analyzed from untreated groundwater samples according to standard methods using membrane filtration with LES Endo agar medium and Chromocult coliform agar medium (SFS 3016 and ISO 9308-1). Spores of sulfite-reducing clostridia were enumerated from water samples after heat treatment of membranes for 15 min at 75°C and incubation for 2 days on tryptose sulfite cycloserine (TSC) agar (ISO 6461-2). Heterotrophic bacteria were enumerated from water samples by the spread-plate technique on Reasoner’s 2 agar (R2A) medium (Difco, USA) and incubated at 22 ± 2°C for 7 days (102, 103).

### Analysis of host-specific MST markers and high-throughput 16S rRNA gene amplicon sequencing.

Total nucleic acids were extracted from DEUF concentrates on membrane filters as described previously in reference 104 using Chemagic DNA plant kit (Perkin Elmer, Waltham, MA, USA). Total RNA was further purified using Ambion Turbo DNA-free DNase kit (Life Technologies, Carlsbad, CA, USA). cDNA was synthesized using Invitrogen Superscript IV VILO system (Thermo Fisher Scientific, Waltham, MA, USA) and used in the 16S rRNA analysis. The total RNA was stored at −75°C or lower, while the cDNA and the DNA extracts were stored at −20°C until use. The gene copy numbers of general fecal indicator *Bacteroidales* bacteria (GenBac3), human-specific fecal indicator *Bacteroides* bacteria (HF183), and Gram-negative bacteria (105) in the samples (including extraction and filtration blanks) were measured from cDNA and DNA extracts as described previously (106). The qPCR assays were performed as described previously (107), by processing 16 μl of RNA in a cDNA synthesis (reverse transcription [RT]). The qPCRs were performed using a QuantStudio 6 real-time PCR system (Applied Biosystems) in 20 μl volume using the TaqMan Environmental PCR master mix (Life Technologies), all with primers and probes at final concentrations 0.2 μM (IDT Technologies, Inc.). The cycling conditions included 95°C for 10 min of enzyme activation and predenaturation followed by 40 cycles at 95°C for 15s of denaturation and at 60°C for 60s of annealing. Standard curves were generated using artificial gene fragments (gBlocks, IDT Technologies, Inc.) containing the sequences for each of the targeted genes. In qPCR, undiluted and 10-fold diluted cDNA and DNA samples were used as qPCR templates to detect PCR polymerase inhibition. For samples in which PCR inhibition was detected, qPCR data were generated using the results from diluted samples. Background signals, if detected in the filtration blanks, were subtracted from all the results to generate the final qPCR and RT-qPCR data per assay. The limit of detection (LOD) was set as 3 copies per reaction. The final qPCR, equivalent LOD (eLOD), and equivalent limit of quantification (eLOQ) values were calculated after taking into account the volume/mass of the processed sample, factors associated with the different processing steps of the RNA and DNA manipulations, and the dilutions used for each sample analyzed.

Subsamples of the nucleic acids were sent to Macrogen Inc. (Seoul, South Korea) for amplicon generation and subsequent sequencing. Bacterial 16S rRNA genes were amplified from DNA (targeting all bacteria present) and cDNA (traditionally considered to target only metabolically active bacteria) using the primers Bakt_341F (5′-CCTACGGGNGGCWGCAG-3′) and Bakt_805R (5′-GACTACHVGGGTATCTAATCC-3′), which target the V3–V4 variable region of the 16S rRNA gene (108). Amplicons were sequenced as 300 bp pair-end reads using the Illumina MiSeq platform. Some samples were sequenced in duplicate to check for reproducibility. Negative controls from different sample processing steps were included in the qPCR and high-throughput amplicon sequencing analysis (tube control for sampling/elution, elution solution, membrane filtration, nucleic acid extraction).

### Sequencing data preprocessing and taxonomic classification.

The 16S rRNA amplicon data for DNA and cDNA libraries were processed and analyzed via the QIIME 2 pipeline (version 2018.11) (109). The DADA2 denoise-paired QIIME 2 plugin was used, with the parameters --p-trim-left-f 9, --p-trim-left-r 9, --p-trunc-len-f 290, and --p-trunc-len-r 250, to trim sequences (to remove bad quality reads with quality score of <20) and to denoise and merge trimmed reads to produce a table of amplicon sequence variants (ASVs) (110). The ASV table was rarefied to a sampling depth of 2,504, which excluded four samples with sequence counts below this threshold (three of these were controls, and one was a duplicate DNA sample). Remaining duplicates were checked for consistency and merged. Taxonomic classification of the ASVs was performed via the q2-feature-classifier plugin in QIIME 2 (111) using a naive Bayes classifier trained on the V3–V4 variable region of representative 16S rRNA sequences. These representative 16S rRNA sequences were derived by clustering 16S rRNA sequences from the SILVA rRNA database (release 132) into operational taxonomic units (OTUs) based on 99% sequence identity (112). The default confidence cutoff of 70% was used in assigning taxonomic labels, as this is designed to balance precision and recall in classifying 16S rRNA sequences (111). Nontarget sequences such as archaeal, mitochondrial, and chloroplastic sequences were filtered out, so that only bacterial sequences remained.

### Assessment of bacterial diversity and bacterial indicator analysis.

Taxonomic classifications of bacterial DNA- and cDNA-based communities were screened for taxa relevant to water safety, e.g., species such as Escherichia coli, Clostridium perfringens, and Pseudomonas aeruginosa and genera such as *Klebsiella*, *Aeromonas*, *Arcobacter*, *Enterococcus*, *Legionella*, *Mycobacterium*, *Yersinia*, and *Listeria*. Alpha diversity metrics for bacterial DNA- and cDNA-based communities, namely, Faith’s phylogenetic diversity, Pielou’s evenness, observed ASVs, and Shannon diversity, were calculated based on the rarefied ASV table using the QIIME2 diversity plugin. Relative abundance values for the bacterial DNA- and cDNA-based communities were calculated in R, and a heatmap showing relative abundance values of selected taxa was generated using the pheatmap function from the pheatmap R package (113).

### Correlation analysis.

Environmental land use data were extracted from maps produced by the National Land Survey of Finland (Maanmittauslaitos) using their online map-viewing tool, *MapSite*. For each of the wells, measurements were made of distances to, and lengths or areas of, nearby roads, fields, marshes, surface water sources, and buildings. “Nearby” land use was defined as land use occurring within 1 km^2^ of the well. In addition to the map-based analyses, site inspections were conducted during sampling visits to examine the immediate surroundings for potential risk factors, and well operators were probed for information on nearby land use and details of any past or suspected problems (e.g., high turbidity, surface water intrusion, fecal indicators). Correlations were sought between physicochemical data, microbial indicator data, environmental land use data, and bacterial alpha diversity data via calculation of Spearman rank-based correlation coefficients using the rcorr function from the Hmisc R package (114). Spearman was used here because Shapiro-Wilk tests carried out in R showed that many parameters were not normally distributed. Correlograms were produced using the corrplot function from the corrplot R package (115). Only statistically significant Spearman correlation coefficients (*P* < 0.05) are reported here, with the following ranges being used for discussion of correlations: very strong (>0.8 or <−0.8), strong (between 0.6 and 0.8, or between −0.6 and −0.8), moderate (between 0.4 and 0.6, or between −0.4 and −0.6), weak (between 0.3 and 0.4).

### Nonmetric multidimensional scaling.

Nonmetric multidimensional scaling (NMDS) plots using weighted UniFrac distance matrices (calculated from the rarefied ASV table in QIIME 2) were created in R using the metaMDS function in the vegan package (116). Environmental variables (e.g., turbidity, dissolved oxygen, nitrate, ammonium) were fitted to the NMDS plots using the envfit function. Only environmental variables that were significantly correlated with community composition (as identified in envfit with a *P* value of ≤0.05) were considered for the figures.

### Data availability.

The 16S rRNA amplicon sequencing data for this study have been deposited in the European Nucleotide Archive (ENA) at EMBL-EBI under primary accession number PRJEB41020 (https://www.ebi.ac.uk/ena/browser/view/PRJEB41020).
