# Supplementary material for: Surface Water Intrusion, Land Use Impacts, and Bacterial Community Composition in Shallow Groundwater Wells Supplying Potable Water in Sparsely Populated Areas of a Boreal Region
Source: Microbiol Spectr. 2021 Nov 3;9(3):e00179-21. doi: 10.1128/Spectrum.00179-21 (PMC8567237; doi:10.1128/Spectrum.00179-21)
Supplement: SUPPLEMENTAL FILE 1 — Supplemental material. Download SPECTRUM00179-21_Supp_1_seq14.pdf, PDF file, 0.1 MB [file spectrum00179-21_supp_1_seq14.pdf]

Supplementary material

Table S1. Spearman rank correlation coefficients showing no significant correlations between environmental data and bacterial alpha diversity

Environmental parameter	Alpha diversity metric	Spearman correlation coefficient	p-value
Total length of nearby roads	Faith's PD (DNA)	0.338961199	0.077647925
Total length of nearby major roads	Faith's PD (DNA)	0.084217168	0.670052308
Total nearby field area	Faith's PD (DNA)	-0.26972674	0.165115085
Total nearby marsh area	Faith's PD (DNA)	0.062944718	0.750331453
Distance to nearest marsh	Faith's PD (DNA)	0.207198579	0.290082935
Total length of nearby ditches	Faith's PD (DNA)	0.150519978	0.444548376
Distance to nearest surface water	Faith's PD (DNA)	0.050410989	0.798916228
Distance to nearest surface water (excluding ditches)	Faith's PD (DNA)	0.137968798	0.483841972
Total nearby surface water area (excluding ditches)	Faith's PD (DNA)	0.097079563	0.623115481
Number of nearby buildings	Faith's PD (DNA)	0.086204959	0.66271321
Total length of nearby roads	Faith's PD (cDNA)	-0.08096644	0.682118124
Total length of nearby major roads	Faith's PD (cDNA)	0.177086784	0.367329493
Total nearby field area	Faith's PD (cDNA)	-0.221738547	0.256784845
Total nearby marsh area	Faith's PD (cDNA)	0.05801861	0.769326798
Distance to nearest marsh	Faith's PD (cDNA)	0.035308609	0.858430326
Total length of nearby ditches	Faith's PD (cDNA)	0.10180624	0.606210787
Distance to nearest surface water	Faith's PD (cDNA)	0.009315074	0.962477873
Distance to nearest surface water (excluding ditches)	Faith's PD (cDNA)	0.199014516	0.30997461
Total nearby surface water area (excluding ditches)	Faith's PD (cDNA)	-0.092666856	0.639067996
Number of nearby buildings	Faith's PD (cDNA)	-0.191353047	0.329344959
Total length of nearby roads	Pielou's evenness (DNA)	0.251682121	0.196366364
Total length of nearby major roads	Pielou's evenness (DNA)	0.221502687	0.257304169
Total nearby field area	Pielou's evenness (DNA)	-0.317163345	0.10006697
Total nearby marsh area	Pielou's evenness (DNA)	0.150519978	0.444548376
Distance to nearest marsh	Pielou's evenness (DNA)	0.07691255	0.69727259
Total length of nearby ditches	Pielou's evenness (DNA)	0.195402299	0.319017265
Distance to nearest surface water	Pielou's evenness (DNA)	0.128219255	0.515542471
Distance to nearest surface water (excluding ditches)	Pielou's evenness (DNA)	0.318094729	0.099019491
Total nearby surface water area (excluding ditches)	Pielou's evenness (DNA)	0.003861119	0.984442524
Number of nearby buildings	Pielou's evenness (DNA)	0.001921767	0.992256343
Total length of nearby roads	Pielou's evenness (cDNA)	-0.026348401	0.894123106
Total length of nearby major roads	Pielou's evenness (cDNA)	0.237077095	0.224477084
Total nearby field area	Pielou's evenness (cDNA)	-0.15058364	0.444353549
Total nearby marsh area	Pielou's evenness (cDNA)	0.105637657	0.592651535
Distance to nearest marsh	Pielou's evenness (cDNA)	-0.066785275	0.735619626
Total length of nearby ditches	Pielou's evenness (cDNA)	0.063492063	0.748229402
Distance to nearest surface water	Pielou's evenness (cDNA)	0.106849379	0.588390711
Distance to nearest surface water (excluding ditches)	Pielou's evenness (cDNA)	0.308239814	0.110528082
Total nearby surface water area (excluding ditches)	Pielou's evenness (cDNA)	-0.114730393	0.56100968
Number of nearby buildings	Pielou's evenness (cDNA)	-0.10624624	0.590509889
Total length of nearby roads	Observed ASVs (DNA)	0.333471949	0.082891537
Total length of nearby major roads	Observed ASVs (DNA)	0.163819696	0.404856147

Total nearby field area	Observed ASVs (DNA)	-0.285722805	0.140508783
Total nearby marsh area	Observed ASVs (DNA)	0.103448276	0.600383683
Distance to nearest marsh	Observed ASVs (DNA)	0.123169565	0.532350412
Total length of nearby ditches	Observed ASVs (DNA)	0.144499179	0.463179871
Distance to nearest surface water	Observed ASVs (DNA)	0.084931558	0.667411262
Distance to nearest surface water (excluding ditches)	Observed ASVs (DNA)	0.19162333	0.328649318
Total nearby surface water area (excluding ditches)	Observed ASVs (DNA)	0.099285917	0.615200622
Number of nearby buildings	Observed ASVs (DNA)	0.129581977	0.511051506
Total length of nearby roads	Observed ASVs (cDNA)	-0.076026115	0.700601747
Total length of nearby major roads	Observed ASVs (cDNA)	0.180547763	0.357887037
Total nearby field area	Observed ASVs (cDNA)	-0.241044142	0.216587883
Total nearby marsh area	Observed ASVs (cDNA)	0.05801861	0.769326798
Distance to nearest marsh	Observed ASVs (cDNA)	0.030655536	0.876936226
Total length of nearby ditches	Observed ASVs (cDNA)	0.106185003	0.590725235
Distance to nearest surface water	Observed ASVs (cDNA)	0.031780841	0.872454554
Distance to nearest surface water (excluding ditches)	Observed ASVs (cDNA)	0.221188073	0.257997963
Total nearby surface water area (excluding ditches)	Observed ASVs (cDNA)	-0.127416926	0.518195596
Number of nearby buildings	Observed ASVs (cDNA)	-0.185038671	0.345851284
Total length of nearby roads	Shannon's diversity (DNA)	0.293949347	0.128941819
Total length of nearby major roads	Shannon's diversity (DNA)	0.230731966	0.237492882
Total nearby field area	Shannon's diversity (DNA)	-0.312750637	0.105142986
Total nearby marsh area	Shannon's diversity (DNA)	0.140120416	0.476982788
Distance to nearest marsh	Shannon's diversity (DNA)	0.087313536	0.658633394
Total length of nearby ditches	Shannon's diversity (DNA)	0.195949644	0.317636729
Distance to nearest surface water	Shannon's diversity (DNA)	0.114520617	0.561730919
Distance to nearest surface water (excluding ditches)	Shannon's diversity (DNA)	0.298932396	0.122282958
Total nearby surface water area (excluding ditches)	Shannon's diversity (DNA)	0.041369132	0.834437543
Number of nearby buildings	Shannon's diversity (DNA)	0.065340065	0.741145331
Total length of nearby roads	Shannon's diversity (cDNA)	-0.055990352	0.777186586
Total length of nearby major roads	Shannon's diversity (cDNA)	0.179970933	0.359450713
Total nearby field area	Shannon's diversity (cDNA)	-0.124107396	0.529209216
Total nearby marsh area	Shannon's diversity (cDNA)	0.04378763	0.824902153
Distance to nearest marsh	Shannon's diversity (cDNA)	0.003831942	0.984560072
Total length of nearby ditches	Shannon's diversity (cDNA)	0.054734537	0.782063878
Distance to nearest surface water	Shannon's diversity (cDNA)	0.048767153	0.805347221
Distance to nearest surface water (excluding ditches)	Shannon's diversity (cDNA)	0.237886677	0.222851606
Total nearby surface water area (excluding ditches)	Shannon's diversity (cDNA)	-0.118039923	0.549687252
Number of nearby buildings	Shannon's diversity (cDNA)	-0.143857958	0.465187961
